# Dynamic of swine influenza virus infection in weaned piglets in five enzootically infected herds in Germany, a cohort study

**DOI:** 10.1186/s40813-024-00390-w

**Published:** 2024-10-01

**Authors:** Kathrin Schmies, Christin Hennig, Nicolas Rose, Christelle Fablet, Timm Harder, Elisabeth grosse Beilage, Annika Graaf-Rau

**Affiliations:** 1 Field Station for Epidemiology, University of Veterinary Medicine Hannover, Foundation, Bakum, Germany; 2 Institute of Diagnostic Virology, Friedrich-Loeffler-Institut, Greifswald-Insel Riems, Germany; 3 Epidemiology, Health and Welfare Research Unit, French Agency for Food, Environmental and Occupational Health and Safety (ANSES), Ploufragan, France; 4 Department of Pathogen Evolution, Helmholtz Institute for One Health, Greifswald, Germany

**Keywords:** Influenza A virus, Weaned piglets, Shedding, Genotypes, Variation

## Abstract

**Background:**

Within the last decades industrial swine herds in Europe grown significantly, creating an optimized reservoir for swine influenza A viruses (swIAV) to become enzootic, particularly in piglet producing herds among newborn, partly immunologically naïve piglets. To date, the only specific control measure to protect piglets from swIAV is the vaccination of sows, which provides passive immunity through maternally derived antibodies in colostrum of vaccinated sows. Interruption of infection chains through management practices have had limited success. This study focused on weaned piglets in five enzootically swIAV infected swine herds in North-West and North-East Germany and aimed to better understand swIAV infection patterns to improve piglet protection and reduce zoonotic risks. Participating farms fulfilled the following inclusion criteria: sow herd with ≥ 400 sows (actual size 600–1850 sows), piglets not vaccinated against influenza A virus and a history of recurrent respiratory problems associated with continuing influenza A virus infection. Influenza vaccination was performed in all sow herds, except for one, which discontinued vaccination during the study.

**Results:**

First swIAV detections in weaned piglets occurred at 4 weeks of age in the nursery and continued to be detected in piglets up to 10 weeks of age showing enzootic swIAV infections in all herds over the entire nursery period. This included simultaneous circulation of two subtypes in a herd and co-infection with two subtypes in individual animals. Evidence for prolonged (at least 13 days) shedding was obtained in one piglet based on two consecutive swIAV positive samplings. Possible re-infection was suspected in twelve piglets based on three samplings, the second of which was swIAV negative in contrast to the first and third sampling which were swIAV positive. However, swIAV was not detected in nasal swabs from either suckling piglets or sows in the first week after farrowing.

**Conclusions:**

Predominantly, weaned piglets were infected. There was no evidence of transmission from sow to piglet based on swIAV negative nasal swabs from sows and suckling piglets. Prolonged virus shedding by individual piglets as well as the co-circulation of different swIAV subtypes in a group or even individuals emphasize the potential of swIAV to increase genetic (and potentially phenotypic) variation and the need to continue close monitoring. Understanding the dynamics of swIAV infections in enzootically infected herds has the overall goal of improving protection to reduce economic losses due to swIAV-related disease and consequently to advance animal health and well-being.

**Supplementary Information:**

The online version contains supplementary material available at 10.1186/s40813-024-00390-w.

## Background

Influenza as a disease entity was first observed in pigs in 1918 and classified as an influenza A virus (IAV) infection in 1930. The disease concurred with the human influenza pandemic known as Spanish flu [1–3]. In the following decades, further IAV subtypes were introduced to and spread in the pig population [4–6]. Nowadays, Asia, America, Europe, Africa and Australia report the presence of at least one of the most common swine influenza A virus (swIAV) subtypes H1N1, H1N2, H3N2 and H1N1pdm09 of which, in Germany, all four are detected [7]. The hemagglutinin (HA) of these subtypes is categorized phylogenetically into clades and subclades as H1 1A “classical swine lineage” (syn. H1pdm), H1 1B “human seasonal lineage” (syn. H1hu) and 1C “Eurasian avian-like lineage” (syn. H1av) [4], while H3 is defined as either “human lineage” (syn. H3hu, derived from human seasonal IAV of 2004/5) or the “swine lineage” (syn. H3sw, derived from human seasonal IAV of the mid 1980s). SwIAV infection can lead to typical clinical symptoms in piglets such as coughing and sneezing, labored abdominal breathing, high fever and lethargy [8, 9] and is a contributor the porcine respiratory disease complex (PRDC). The grade of clinical signs of swIAV infection varies from subclinical over mild to severe, and even fatal courses have been reported. Among other factors, the course of the disease is influenced by the dose and virulence of the virus [10, 11]. Moreover, the immunological status of the infected pig as well as possible co-infections with bacterial or other viral pathogens are modulating clinical signs [12, 13].

Although clearly secondary in economic impact to epidemic transmissible infections such as classical swine fever virus, swIAV infections can cause substantial and, in particular, continuing economic losses due to impaired reproduction and increased morbidity leading to reduced growth performance and rising costs for vaccination and (antibiotic) treatment of co-infections [14, 15].

The increased number of pigs within a herd and the high turnover of immunologically naïve animals are important factors in creating an optimized reservoir for enzootically infected farms [16, 17]. Piglets are suspected drivers of swIAV infection dynamics as there is a continuous influx of susceptible newborns; their propensity for developing swIAV clinical signs is highly modified by the level of maternally derived antibodies (MDA) [12, 18].

Colostrum production by the sow as well as colostrum intake by the individual piglet, however, is highly variable. Therefore, the time an individual in a group of piglets is becoming susceptible to swIAV infection also varies considerably. These difficult-to-harmonize situations supports ongoing virus circulation in the group [19, 20]. In addition to epidemic outbreaks, enzootic courses of swIAV infection occur in pig herds [21]. In general, an enzootic course of disease is characterized by a stable and predictable prevalence and mortality is rated to be very low [22]. Here, we refer to enzootic swIAV infections in pig herds as a detectable presence of swIAV that continues over extended periods of months and even years. This circulation is independent of new viral incursions from outside but is based on self-sustaining virus amplification on the farm, which is made possible by the continuous supply of fully susceptible host individuals (newborn piglets). Self-sustaining swIAV circulation in such farms enhances the risk of antigenic drift and, if new swIAV strains enter the holding, foster reassortment events potentially leading to new genotypes [23–25]. High antigenic variability challenges efficacy of vaccines and control strategies for farmers and veterinarians. In addition, phenotypical variation of swIAV may include increased zoonotic propensity and, hence, raises public health concerns. So far, vaccination of sows with either commercial licensed or autologous adjuvanted whole inactivated vaccines remains the most frequently used specific control strategy for swIAV in sow herds [26], while concepts to effectively prevent younger pigs against swIAV or even allow to interrupt the virus circulation are still lacking. The number and location of N-linked glycosylation sites in the hemagglutinin can influence the accessibility of neutralizing antibodies. Hence, N-linked glycans attached to critical sites can shield neutralizing epitopes and thus may foster the emergence of virus variants that escape vaccination-induced immunity [27].

This cohort study focused on a better understanding of the dynamic of swIAV infections in weaned piglets in German pig herds enzootically infected with swIAV (please refer to Additional file 1: Table S1). Increased knowledge of the infection dynamics of swIAV in enzootic herds should help to find more specific starting points for improved prevention, i.e. re-definition of the optimal piglet age to start active vaccination. This in turn helps to reduce economic losses due to swIAV-related disease and consequently to advance animal health and well-being.

## Results

### Production characteristics of the five study herds

Five pig herds (A-E) fulfilling the inclusion criteria laid down in the materials & methods section were selected out of twelve herds scrutinized and were further characterized using a questionnaire: Herds A-E are conventional, high-performance breeding to nursery herds in the North-West and North-East of Germany. Numbers of sows in each herd range from 600 to 1850. Nurse sows are used in herd A, B and E. Only herd E does not clean and disinfect farrowing rooms between batches. While herd A discontinued sow vaccination during the study period, herds B to E used two commercial vaccines (Respiporc® FLU3 and Respiporc® FLUpan H1N1, Ceva Santé Animale, France) for the sow herd vaccination (please refer to Table 1). Growing pigs were not vaccinated in any of the herds. Self-replacement of gilts was practiced in herd A and B, whereas herds C to E purchased gilts from an external source. Duration of quarantine (5–8 weeks), cleaning and disinfection (C&D) between batches as well as the age of gilts (4–6 months) at swIAV vaccination differed between herds. The piglet rearing period ranges from 6 to 8 weeks and for herd E, 1 to 3 age groups were kept per compartment. C&D of nursery compartments was performed in all herds (Table 1).

**Table 1:** Productions characteristics of five breeding-to-nursery herds based on a questionnaire survey

Parameter	Herd A	Herd B	Herd C	Herd D	Herd E
Neighborhood, pig density*	High	Low	High	High	High
Sow herd					
Herd size (sow number)	1800	1850	700	600	700
Batch farrowing interval (weeks)	1	1	4	4	3
Suckling period (weeks)	4	4	3	3	4
Nurse sow system	Yes	Yes	no	No	Yes
C&D* between batches	Yes	Yes	Yes	Yes	No
Sow herd influenza A vaccination interval	None	4 months	3 months	4 months	3 to 4 months
Influenza A vaccine	None	Respiporc® FLUpan, Ceva Santé Animale, France Respiporc® FLU3,Ceva Santé Animale, France			
Gilt management					
Self-replacement	Yes	Yes	No	No	No
Quarantine location	Not required	Not required	Separate building	Separate building	Separate building
Quarantine period (weeks)	6	Not required	5	8	6
C&D* between batches	Yes	Not required	No	Yes	Yes
Influenza A vaccination (age)	None	4 and 5 months	6 months	6 and 7 months	6 and 7.5 months
Influenza A vaccine	None	Respiporc® FLUpan, Ceva Santé Animale, France Respiporc® FLU3, Ceva Santé Animale, France			
Nursery					
Rearing period (weeks)	6–7	7	7	7–8	6–7
Age groups per compartment	1	1	1	1	1–3
Density change	No	No	Yes	Yes	Yes
Pen size at weaning	65	24	90	300	90
C&D** between batches	Yes	Yes	Yes	Yes	Yes
Ventilation	Fresh air from outside via attic through ceiling and sheath; exhausted air outlet through the top	Fresh air from outside via service corridor through ceiling; exhausted air outlet underfloor	Fresh air from outside via service corridor through ceiling; exhausted air outlet through the top	Fresh air from outside via service corridor through ceiling and sheath; exhausted air outlet underfloor	Fresh air from outside via service corridor trough ceiling and sheath; exhausted air outlet through the top
Influenza A vaccination	None	None	None	None	None
PRRSV herd status (according to Holtkamp et al.,2021 [28])	Category II-vx: positive stabile with vaccination	Category IV: negative	Category II-vx: positive stabile with vaccination	Category I-B: positive unstable, low prevalence	Category II-vx: positive stabile with vaccination
PRRSV vaccination	Piglets and sows	None	Piglets, gilts and sows	Piglets, gilts and sows	Piglets, gilts and sows
Porcine Circovirus 2 vaccination	Piglets	Piglets and gilts	Piglets and gilts	Piglets and sow	Piglets and gilts
* Mycoplasma hyopneumoniae* vaccination	None	Piglets and gilts	Piglets	Piglets	Piglets and gilts
* Actinobacillus pleuropneumoniae* vaccination	None	None	Gilts	Gilts	Gilts
* Bordetella bronchiseptica* vaccination	Sows	None	None	None	None
* Streptococcus suis* vaccination	Sows	None	None	Sows	None

*Pigs kept per 100 hectares of land in the herd’s district [29]

Very low: 0–64; low: > 64–169; moderate: > 169–406; high: > 406–862; very high: > 862–1986

**C&D: cleaning and disinfection

### SwIAV detection concentrated in the nursery

**Fig. 1:**
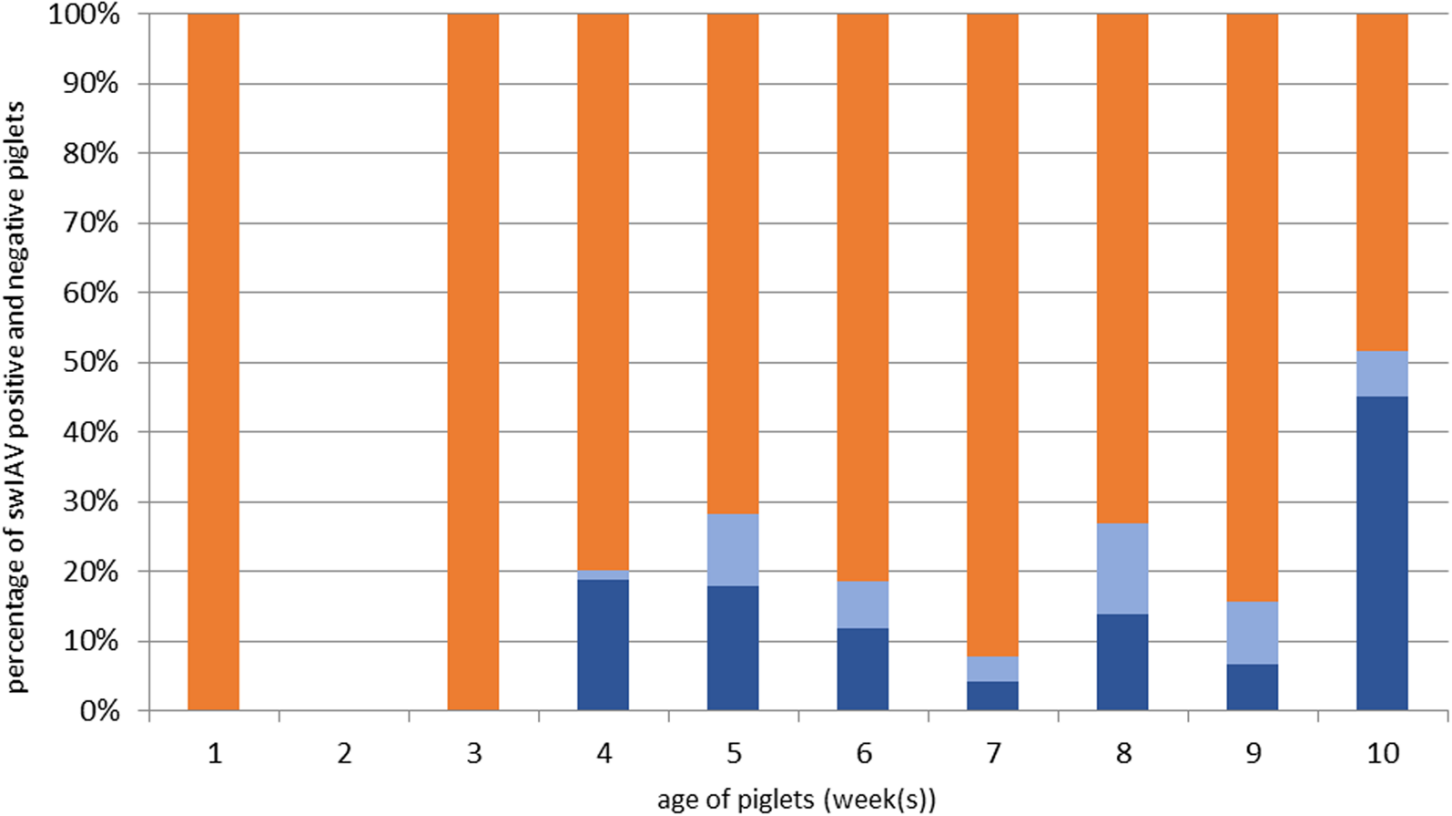
Merging the detection of swIAV RNA loads (cq ≤ 35, dark blue; cq > 35, light blue) by RT-qPCR in nasal swabs of piglets 4 to 10 weeks of age of the five selected study herds. Orange columns depict negative RT-qPCR for swIAV

A total of 1,370 nasal swabs from piglets was examined; 360 nasal swabs were from suckling piglets 1 week of age, 60 from suckling piglets 3 weeks of age and 950 from weaned pigs 4 to 10 weeks of age. Nasal swabs PCR-positive for swIAV (n = 216) were only found in weaned pigs housed in the nursery, while all nasal swabs collected from suckling piglets were PCR-negative for swIAV (Table 2, Fig. 1). In addition, nasal swabs collected from sows belonging to these piglets in the first week after farrowing were also negative.

**Table 2:** Detection of swIAV in nasal swabs of piglets in study herds A-E by RT-qPCR at different weeks of age

	Age (week(s))	Piglets sampled (n)	Herd tested PCR-positive (cq ≤ 35)	Herd tested PCR-negative
Suckling piglets	1	360	None	A, B, C, D, E
	2	0	–	–
	3	60	None	B
Weaned piglets	4	69	**E**	A
	5	252	**A, B, C, E**	D
	6	135	**A, B, E**	None
	7	140	**D**	B, C
	8	160	**A, C, E**	B
	9	134	**A, D**	C
	10	60	**A**	C

A herd with at least one positive sample (cq ≤ 35) is marked in bold

The age swIAV was first detected in the weaned pigs differed between herds. In herd E, swIAV was first detected in weaned pigs at 4 weeks of age. Sampling in weaned pigs at 5 weeks of age revealed swIAV infection in 4 herds (herd A, B, C, E). In herd D, swIAV infection in piglets was confirmed at 7 weeks of age (Table 2). Considering all herds and the entire observation period, swIAV was found during the entire nursery period. Highest swIAV percentage in weaned pigs was found at 4 (18.8%) and 10 weeks of age (45%), while percentages in piglets 5 to 9 weeks of age ranged from 4.2 to 17.9% (Fig. 1).

In the longitudinal sampling, 116 of all 360 piglets (32.2%) showed higher swIAV loads at least once (cq ≤ 35). In a total of 18 weaned pigs (5%), higher swIAV load was detected twice (cq ≤ 35) at two distinct time points. The affected piglets were 4 and 6 weeks, including 13 days between detections (n = 1), 5 and 6 weeks, including 7 days between detections (n = 4), 5 and 8 weeks of age, including 19 days between detection (n = 10) or 9 and 10 weeks of age (n = 2). For another two weaned pigs (0.5%) higher swIAV load was detected on three consecutive sampling days (cq ≤ 35). The affected piglets were 5, 6 and 8 weeks of age (Table 4).

### Viral RNA load measured by cq

In the nasal swab samples positive for swIAV (n = 216) including samples with cq ≤ 35 and samples with cq 35.1–39.9, 63.9% had a cq ≤ 35 (mean value and standard deviation 31.7 ± 4.4) indicating moderate to high virus loads, and 36.1% a cq > 35 (mean value and standard deviation 35.8 ± 1.3) indicating lower virus loads excreted that are less likely to be effective in virus transmission. Percentage of detection viral RNA load with cq > 35 range from 1.4 (4 weeks of age) to 13.1% (8 weeks of age). Samples with a cq ≤ 35 were the most frequent finding in piglets 4 to 10 weeks of age, except in piglets 9 weeks of age (Fig. 1).

### Distinct swIAV subtypes circulated in piglets up to 10 weeks of age in different holdings

In the majority (n = 118, 54.6%) of positive nasal swabs, swIAV could be fully subtyped (Table 3). The most frequently detectedsubtype was H1avN1 (39.8%), followed by H1pdmN2 (11.0%) and H1avN2 (4.6%). H1huN2 (0.9%) was detected only sporadically, and no evidence for subtype H3 infection has been found. However, in a total of 98 (45.4%) samples positive for swIAV, full subtyping was not possible. The missing information is characterized by Hx or Nx (Table 3). In samples with successful subtyping, the mean cq and standard deviation was 27.5 ± 4.2 and the median was 27.3, while the mean cq and standard deviation was 36.4 ± 2.0 and the median was 36.4 in samples that could not be fully subtyped. There was no statistical confirmation (*p* = 0.93) between low viral loads and subtyping failure. The simultaneous circulation of two different subtypes was detected in herd E—H1avN1 and H1pdmN2—in piglets 6 weeks of age and in herd D—H1avN1 and H1avN2—in piglets 7 weeks of age. In a total of 11 piglets also the second positive sample could be subtyped, and the same subtype emerged in each case (Table 4). For 7 further piglets, the subtype could only be determined incompletely for one sampling. For the remaining 2 piglets, a change in hemagglutinin could be determined from the first to the second detection (Table 4).

**Table 3:** Frequency of swIAV subtypes detected in weaned piglets with a PCR-positive nasal swab (n = 216) from five enzootically infected herds

Subtype	Proportion (%)
H1avN1	39.8
H1avN2	4.6
H1pdmN2	11.0
H1huN2	0.9
H1avNx	2.3
H1pdmNx	2.3
HxN1	3.2
HxN2	3.2
HxNx	34.3

Hx/Nx: the segment cannot be subtyped

**Table 4:** Prolonged observed shedding of moderate to high swIAV loads (cq ≤ 35) in individual pigs

Piglet	Herd	Age (weeks) at 1st swIAV detection (Cq value)	Age (weeks) at 2nd swIAV detection (Cq value)	Age (weeks) at 3rd swIAV detection (Cq value)	Time between detections (days)	Subtype detected for 1st detection	Subtype detected for 2nd detection	Subtype detected for 3rd detection
1	E	4 (cq 24.6)	6 (cq 26.8)	–	13	H1pdmN2	H1pdmN2	–
2	E	5 (cq 21.1)	6 (cq 30.0)	–	7	H1avN1	H1avN1	–
3	E	5 (cq 26.5)	6 (cq 28.1)	–	7	H1avN1	H1avN1	–
4	E	5 (cq 34.8)	6 (cq 25.9)	–	7	HxNx	H1avN1	–
5	E	5 (cq 25.8)	6 (cq 30.8)	–	7	H1avN1	H1avN1	–
6	E	5 (cq 21.1)	8 (cq 26.8)	–	19	H1avN1	H1avN1	–
7	E	5 (cq 33.6)	8 (cq 25.1)	–	19	H1avN1	H1avN1	
8	E	5 (cq 33.6)	8 (cq 30.7)	–	19	HxNx	H1avN1	–
9	E	5 (cq 34.2)	8 (cq 31.9)	–	19	HxNx	H1avN1	–
10	E	5 (cq 25.0)	8 (cq 31.1)	–	19	H1avN1	H1avN1	–
11	E	5 (cq 34.0)	8 (cq 21.8)	–	19	H1xNx	H1avN1	–
12	E	5 (cq 31.3)	8 (cq 22.8)	–	19	H1avN1	H1avN1	–
13	E	5 (cq 24.4)	8 (cq 35.0)	–	19	H1avN1	HxNx	–
14	E	5 (cq 22.5)	8 (cq 28.2)	–	19	H1avN1	H1avN1	–
15	E	5 (cq 21.2)	8 (cq 26.8)	–	19	H1avN1	H1avN1	–
16	A	9 (cq 34.5)	10 (cq 27.4)	–	7	H1pdmNx	H1avN1	–
17	A	9 (cq 34.4)	10 (cq 32.2)	–	7	H1pdmNx	H1avN1	–
18	A	9 (cq 32.8)	10 (cq 32.0)	–	7	H1avNx	H1avN1	–
19	E	5 (cq 27.0)	6 (cq 32.0)	8 (cq 23.7)		H1avN1	H1avN1	H1avN1
20	E	5 (cq 25.2)	6 (cq 34.2)	8 (cq 27.3)		H1avN1	HxNx	H1avN1

### Evaluation of passive antibody levels in piglets at one week of age and exhibition of high levels of swIAV shedding

Most piglets (98.3%) and most of the according sows (95.0%) tested positive for antibodies specific for the nucleoprotein (NP) of swIAV. In 88.0% of the piglets the S/P ratio ranged from 0.4 (threshold distinguishing seropositive and -negative) to 11.8, while 10.0% showed exceedingly high positive S/P ratios above 2000. However, a tendency that swIAV was detected more frequently at higher viral loads in piglets showing lower S/P ratio levels at birth was statistically not supported (*p* = 0.55 for S/P ratios 0.4–11.8 and *p* = 0.64 for S/P ratios > 2000) (Table 5). Although there is no significant association between the S/P ratio of blood samples from piglets one week of age and the probability of detecting swIAV via PCR in nasal swabs, there are farm differences regarding the probability of swIAV PCR positive results.

**Table 5:** Detection of swIAV in nasal swabs of weaned piglets according to their S/P ratio of swIAV specific serum antibodies at one week of age

S/P ratio*	Piglets (n)	Percentage of swIAV positive** piglets (%)
0.0–0.3	6	33.3
0.4–11.8	318	64.7
> 2000	36	27.8

*S/P ratio ≥ 0.4 is regarded positive; ** RT-qPCR, cq ≤ 35

Statistical analysis based on mixed logistic regression

### Long term presence of swIAV infections in herds A-E

**Fig. 2:**
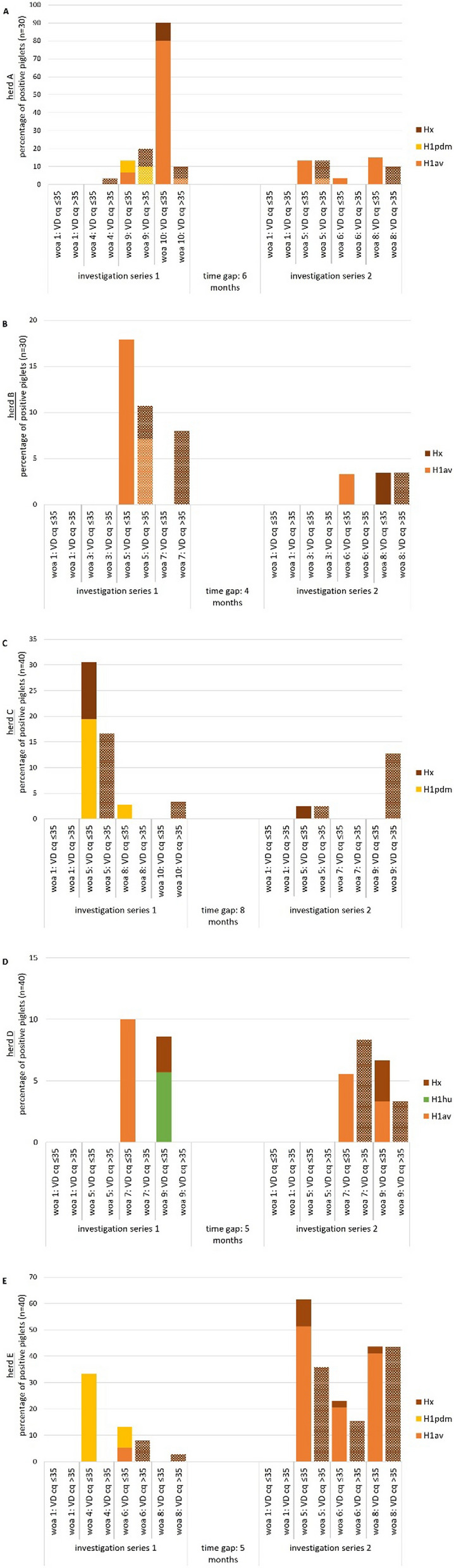
Continuing presence of swIAV infections in five herds **A**–**E** at different weeks of age (woa) detected in two investigation series separated by 4–8 months, VD: viral detection, detection of subtypes with cq > 35 is shown hatched. n = 30 piglets tested for herd A and B, n = 40 piglets tested for herds ** C** to ** E**

SwIAV infections have been detected in all five herds in both investigation series. This means that swIAV infections have either been present over prolonged time periods or continuous re-introduction of swIAV was detected during the study period of at least 8 to 13 months. During this study, the same swIAV subtypes were detected in the individual herds over the entire period as already previously reported (Additional file 1: Tables S1 and S3). Percentage of viral detection at higher virus loads (cq < 35) was highly variable within herds ranging from 5% (herd C, 5 weeks of age) to 90.0% (herd A, 10 weeks of age). Considering also cq values > 35, percentage of 100.0% (herd A 10 weeks of age) and 97.5% (herd E, 5 weeks of age) could be detected. Except for herd E, viral detection rates were lower in the second investigation series (Fig. 2).

For a total of nine swIAV positive samples from the five selected pig herds, whole genome sequencing was performed (Table 6; Additional file 1: Table S2). Due to low viral loads (cq > 32), sequencing of two further viruses (herd C, investigation series 2 and herd D, investigation series 2) failed. Five samples (of herds A, B, C and E) could be fully genotyped yielding four previously described genotypes: A, AQ, AT, and R [30]. Four samples of herds B, D and E could not be assigned to a specific genotype (undetermined) since for some segments only partial sequences were obtained (Table 6). However, the HA of the nine sequences could be assigned unequivocally to three H1 clades: 1A.3.3.2/pdm (II-like), 1B.1.2.1 or 1C.2.2 (Table 6).

**Table 6:**
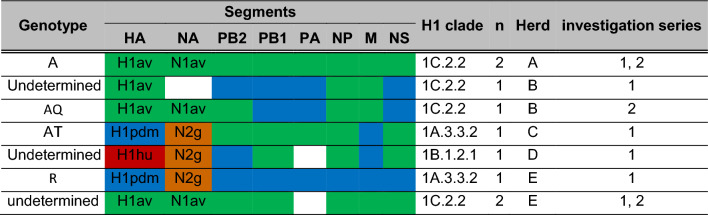
Genotypes of whole genome sequences of swIAV from pig herds A-E

Genotype nomenclatures were assigned as described previously by Graaf-Rau et al., 2023 [30]

The color coding indicates phylogenetically distinct lineages for each segment. The origin of the internal gene segments PB2 to NS is assigned to avian (green color) or human pandemic 2009 (pdm, blue) origin. N2g indicates closest relationship with A/swine/Gent/1/1984-like sequences. White boxes indicate lack of full sequences, hampering a clear designation of the genotype.

Sequences of both investigation series of herds A and B clustered in clade 1C.2.2. The sequences from the first investigation series of herds C and D belonged to clade 1A.3.3.2/pdm (II-like) and clade 1B.1.2.1, respectively, confirming RT-qPCR results that two viruses were circulating on these holdings. Unfortunately, due to low viral loads, neither the subtype (RT-qPCR) nor the sequence of swIAV positive samples could be obtained for the second investigation series of these two herds. Also, for herd E two different subtypes were detected in the first round of investigations. One sample of each subtype was sequenced and could be assigned to clade 1C.2.2, the other one clustered with 1A3.3.2/pdm (II-like), respectively. For investigation series 2, only viruses belonging to clade 1C.2.2 were found.

Neutralization-relevant epitopes within the HA1 fragment of hemagglutinin are critical regions targeted by by neutralizing antibodies as important effectors of a protective immune response. Thus, these regions are key targets for vaccine development. However, even single amino acid substitutions in these regions can hamper and even abrogate the binding of neutralizing antibodies leading to so-called immune escape. Therefore, amino acids (AA) predicted to be located in these HA1 epitopes [30, 31] were compared between virus sequences from investigation series 1 and 2 of a herd. This analysis helped determine if the sequences were derived from the same clade (Additional file 1: Table S3). 1–3 AA substitutions in investigation series 2 sequences were detected in viruses from herds A, B, and E [30, 31]. These included Sa position 125, n = 1 (herd A), position 155, n = 1 (herd E), Sb position 185, n = 1 (herd A), position 190, n = 1 (herd A) and Ca2 position 135, n = 1 (herd B). In addition, in investigation series 2 of herd B, two potential additional N-linked glycosylation sites have been predicted that might be involved in shielding neutralization-relevant epitopes (Additional file 1: Table S4).

## Discussion

In farrow-to-finish herds the continuous introduction of young pigs that are susceptible to infection despite MDA has been claimed a major driver towards an enzootic swIAV status [31]. In the five farms investigated here, selected i.e. because of a history of enzootic swIAV infections, all nasal swabs from sows in the week after farrowing as well as all nasal swabs from suckling piglets collected at the same time, tested negative for swIAV. Based on these results, we conclude that in the study herds transmission of swIAV from the sow to the piglets did not occur during the first week of age. However, with the selected sample key (n = 30 or 40 piglets) per herd, it must be considered—even if quite unlikely—that a swIAV infection prevalence below 10% cannot be excluded, even if swIAV was not detected in nasal swabs. In contrast, however, a cross-sectional study by Lillie-Jaschniski et al., 2022 [32] revealed swIAV infection in suckling piglets at 1–4 weeks of age in enzootically and epidemically infected farms in Europe. In addition, an enzootically infected herd in Denmark showed high swIAV prevalence in suckling piglets of 1–5 weeks of age and swIAV infection in nasal swabs of the unvaccinated respective sows [24]. Although no swIAV was detected in sows in the present study, the literature indicates that sows can be an important source of transmission of swIAV to piglets [24, 33]. An important factor found here to influence early swIAV transmission is the vaccination status of the sow herd. With the exception of herd A, that stopped vaccination during the study period, all sows and gilts of the other study herds have been vaccinated using both commercially available, licensed vaccines (Respiporc® FLUpan (H1pdmN1) and Respiporc® FLU3 (H1avN1, H1huN2, H3N2), Ceva Santé Animale, France). Although highly speculative, it cannot be excluded that the sow herd immunity induced by vaccination against swIAV at intervals of 3 to 4 months might have reduced the sow-to-piglet transmission rate in this study. Yet, the aforementioned cross-sectional study did not reveal a significant influence of sow herd vaccination on virus detection in the piglets [32]. Vaccination not only aims at lowering the risk of swIAV transmission from the sow to the piglets but also at protecting piglets clinically by MDA received with the colostrum. However, infection and shedding of swIAV was not prevented by vaccination alone [12, 18, 34].

Another factor that might have prevented early transmission of swIAV between sows and piglets in this study is the strict all-in-all-out management in the farrowing rooms that was implemented in all herds except herd E (please refer to Table 1). However, transmission between sows and piglets was not detected in herd E either. Keeping piglets at different age groups in the farrowing unit has been identified as a promoter for younger piglets to become infected [19]. Other factors known to influence swIAV infection of sows and the virus transmission to suckling pigs are gilt acclimation practices and pig density in the neighborhood [35, 36]. Due to the small sample size of five herds included in this study and (intended) extensive similarities in the herd characteristics and management practices respective risk factors cannot be statistically analysed here.

In this study, swIAV was earliest detected after weaning. Sampling during the suckling period (one week of age (herds A-E) and three weeks of age (herd B)) remained negative for swIAV. The youngest weaned piglets positive for swIAV were 4 weeks of age. During the nursery period the percentage of swIAV infection was highest at 4 and 10 weeks of age and lower in the time between. The detection of swIAV at several time points during the nursery period and over the entire study period of 8–13 months in two investigation periods is suggestive of an enzootic infection. It is well known, that large herds with a batch farrowing system, as it was implemented in the study herds, have a constant influx of swIAV susceptible piglets into the population which is, according to mathematical modelling, a prerequisite for an infection to become enzootic [17, 37–39]. In the aforementioned cross-sectional study, swIAV infections were detected significantly more often in younger weaned piglets (4 to 6 weeks of age) compared to older piglets (7–9 weeks of age) [32]. The higher detection rate in the first week after weaning is likely influenced by the mixing of piglets from different litters. In our study herds, the weaned piglets were assorted by weight and sex and co-mingled to groups of 24 to 300 piglets per pen. Large groups and a space limited to 0.35 m^2^ per piglet inevitably leads to more frequent direct animal contact. Higher numbers of infected piglets might also lead to increased aerosolization of swIAV with heightened risks of wider virus dissemination across different pens within the holding [40, 41]. However, the prevailing routes of virus dissemination remain unresolved.

In this respect, swIAV infection is influenced by climate control, including temperature, humidity and ventilation profile but also air flow between rooms with piglets of different age groups must be considered as a possible source of infection [21, 42, 43].

Real time RT-PCR has become the routine diagnostic tool for detecting swIAV infections [30, 44]. Nonetheless, some limitations and pitfalls regarding the interpretation of the results need to be considered. Different from other matrixes, like blood or tissue, nasal swabs are vulnerable to compromised sampling procedures. The swab must be inserted deep into the nose without injuring the ethmoid and the swab must be given enough time to soak up a sufficient amount of nasal secretions [45]. In this study, the time was set to 9 s combined with a one third turn every three seconds. With this procedure we tried to harmonize sampling procedures and ensure to gain enough material from the nasal mucosa. Yet, air or dust, containing viral RNA, inhaled shortly before sampling may lead to a positive PCR result falsely being interpreted as evidence of productive infection. As such, dust contamination is expected to yield low viral RNA loads and cq values > 35 were interpreted with caution regarding a discrimination of infection versus contamination of the upper respiratory tract. Similar findings clearly demonstrating a contamination were made for *Mycoplasma hyopneumoniae* detected in the upper respiratory tract in farm personnel that stayed in pig herds for a certain amount of time [46]. However, a fading or incipient productive swIAV infection can also present cq values > 35. Nonetheless, low virus loads as indicated by high cq values are supposedly less important for the infection dynamics within a pig herd. Here, the threshold value differentiating samples likely relevant for the dynamic of the infection within the group of pigs and those likely not was arbitrarily set to a cq ≤ 35. The comparatively small section of pigs showing an enhanced amount of viral load in nasal swabs (10.1%) supports the assumption of a low-level enzootic swIAV infection in the study herds and is in accordance with previous findings [19, 47–49]. The low prevalence of highly effective virus shedders might be related to the presence of MDA homologous to the circulating virus strain [50]. Interpreting the results, it should be considered that the detection of the same subtype over longer time is a strong indicator for an enzootic course of the infection but not the final proof. Alternatively, a frequent re-introduction of the virus from an external source leading to a constant sequence of epidemics has to be discussed.

Nearly all sows and piglets were seropositive for nucleoprotein (NP) specific antibodies in this study. High prevalence of antibodies against swIAV in sows and piglets are also described in other studies [51, 52]. Even though it is not possible to differentiate antibodies derived from either infection or maternally by an NP-specific ELISA [53, 54], antibodies detected in piglets in the first week of age are generally assumed to be maternally derived, as very early infection that can occur within the first three days of age [19] would at least need 10–14 days until antibodies as part of an active immune response can be detected. The protective effect of MDA produced by vaccination of the sow against swIAV infection is debatable [18, 47, 55, 56]. Although this study did not find a statistically significance between the level of maternal antibodies, measured by NP-specific S/P ratio, at one week of age and swIAV infection, it is well known that MDA cannot fully protect piglets from infection [12] but from clinical disease [18]. It is also known that MDA can impair the active immune response (IgG, IgA, T-cells) to a swIAV infection in a way that impedes a fully resilient immunity [12, 18]. Those piglets become susceptible to infection with the same swIAV subtype again within a short time [12, 18, 55, 56].

For three of the study herds (A, B and E) the same swIAV H1, clustering in clade 1.C.2.2, was found in the first and second investigation series. For the analysed sequences of these herds at least 1–3 AA substitutions were detected in sites identified to be relevant for binding of neutralizing antibodies. Considering the pause between the two investigation series of four to eight months, detecting the same subtype and even genotype (herd A and E) is a strong indicator that swIAV persists in these herds. This is in accordance with previous findings by Ryt-Hansen et al., 2020 [24], detecting the same subtype (H1avN2) over a one-year period with a monthly sampling interval. In addition, the same study identified a high substitution rate for the HA gene comparable of that to human influenza virus [24]. Closer monitoring in the farms and an increased number of sequenced samples would be necessary to make a prediction about the substitution rate here. Nevertheless, the mutations found here in antigenic sites of the HA gene and additional N-linked glycosylation sites (herd B) are suggestive of antigenic drift within the study period [57]. For herds C and D it is impossible to state, whether the viruses in investigation series 1 and 2 were the same or if new viruses of similar subtypes entered the herds at the second time point of investigations due to low viral loads in the second investigation series. Understanding variability of neutralization-relevant epitopes is also crucial for designing effective influenza vaccines. Since these sites are prone to mutations that can lead to immune escape, vaccines need to induce a broad and robust immune response that targets multiple epitopes. This knowledge helps in developing vaccines that are more likely to remain effective despite the antigenic drift of the virus.

Co-circulation of different swIAV subtypes was found in two study herds. In herd E, H1pdmN2 (clade: 1A.3.3.2) and H1avN1 (clade: 1C.2.2) was found at the same sampling in the first investigation series but in different individuals at the age of 6 weeks. Unfortunately, the genotype of the 1C.2.2 clade for herd E remained undetermined, so that no conclusion can be made about reassortment of these viruses. In herd D, a co-infection with H1avN1 and H1avN2 was detected in the first investigation series in two piglets aged 7 weeks. Co-circulation as well as co-infection of different swIAV subtypes in pigs has been described previously [25, 32, 58, 59].

One of the characteristics of swIAV is a shedding period up to 7–10 days post infection [60]. Compared to porcine reproductive and respiratory syndrome virus (PRRSV) or porcine circo virus 2 (PCV2) infections [61, 62] the time of swIAV shedding is quite short. In this study 18 piglets were tested positive on two (consecutive) samplings spaced for up to 19 days and two piglets were tested positive on three consecutive samplings. SwIAV shedding is influenced by the presence of MDA with higher levels associated with longer shedding in individuals [18, 59]. However, re-infection with the same swIAV strain should also be considered [19, 47] when individuals are tested positive for longer time periods. In addition, we do not have contiguous data about the PRRSV, PCV2, or *M. hyopneumoniae* status of the animals which also might have modulated shedding patterns. In cases of re-infection, sequencing of virus strains might help to distinguish prolonged shedding from re-infection. Re-infection with the same subtype might also influence the course of enzootic swIAV infections in groups of weaned piglets as can be concluded from the results gained from ten piglets of this study: These piglets first tested positive and in the subsequent sampling negative before they became positive again with the same subtype in the third sampling. Waning MDA in weaned piglets might enhance the risk for re-infection in weaned piglets [12, 56].

## Conclusion

In conclusion, long term presence of swIAV in five breeding herds (farm sizes: 600–1850 sows) included in this study focused on weaned pigs over the entire nursery period. Sources of infection of weaners, thus, might be independent from sow-suckling piglet contacts. SwIAV infections in groups of nursery piglets are known to be driven by a high number of susceptible piglets kept in a compartment. Here prolonged virus shedding by some individual piglets or even re-infections as well as co-circulation of different swIAV subtypes in a group or even individuals complicated the course of infection in larger populations of weaned piglets from four to ten weeks of age. Prevention of swIAV circulation in enzootically infected herds essentially requires interruption of virus transmission within the groups of weaned piglets. In addition, the prevention of swIAV infections in pig herds requires the interruption of transmission chains between groups of different compartments and/or different ages through e.g., contaminated fomites and aerosols. These piglets still may present with (waning) titers of MDA. The main burden of swIAV infections was detectable in piglets after weaning, especially in weeks 4 and 10. Therefore, it seems reasonable to develop vaccines and vaccination strategies that can guarantee a robust active immunity already at this stage of production.

## Methods

### Study design

The cohort study was carried out from October 2021 until November 2023 within the framework of the European collaborative project PIGIE (ICRAD: 2821ERA24). In six European countries (France, Spain, Italy, Denmark, Great Britain, Germany) conventionally kept pig herds were monitored in parallel. In Germany, five participating herds (herd A to E) were selected by the following criteria: farmer willing to participate, sow herd with ≥ 400 sows, piglets not vaccinated against influenza A virus, history of recurrent respiratory problems associated with continuing influenza A virus infection. The study design foreseeing the repeated invasive sampling of pigs was approved by ethics committees as listed in the declaration below.

A sampling schedule was followed in each herd in two separate investigation series comprising each a sample of ten randomly selected sows and three to four of their piglets, also randomly selected (please refer to Table 7). Of the average 16 piglets in a litter, four medium-sized, vital, clinically inconspicuous piglets per litter were selected for the study. The sows were selected according to the piglets, whereby care was taken to ensure that only healthy sows were selected. The sampling started in the week after birth and ended when the piglets left the nursery unit. Within this time, piglets were sampled four times and the sows once (Table 7). Sampling in the first week after birth was mandatory; the other three dates were selected according to the occurrence of clinical swIAV symptoms during the study and the occurrence of typical swIAV infections based on previous experience of the farmer and veterinarian. The piglets were individually ear-tagged to ensure follow-up identification until the end of the study period. Because of baseline piglet mortality and loss of ear tags, the number of sampled piglets was increased from 30 to 40 for the sampling in herds C to E. Nasal swabs from piglets in their first week of age were taken by the use of Sigma-Virocult Mini® swabs (Polyurethan tip, 1.0 ml, Check Diagnostics GmbH), while sampling of older piglet was done with Sigma-Virocult® swabs (Polyurethan-HNO tip, 1.0 ml, Check Diagnostics GmbH). Nasal swabs were taken by the veterinarians carrying out this study, inserting the swab at least 4 cm into each nostril for 9 s combined with a one third turn every three seconds. Blood samples from piglets were collected twice by puncture of the jugular vein, from sows once (Table 7) employing the Monovette system (Sarstedt, Germany). The same sampling schedule was repeated in each herd after four to eight months.

**Table 7:** Samples and sampling schedule

Sampling	Nasal swab* (piglets)	Nasal swab (sows)	Serum (piglets)	Serum (sows)
1	x	x	x	x
2	x	–	–	–
3	x	–	–	–
4	x	–	x	–

*Sample size: herd A and B n = 30 piglets, herd C to E n = 40 piglets

### Investigation site

Samples were individually tested at the National Reference Laboratory for Avian Influenza of the Friedrich-Loeffler-Institute (FLI), Greifswald-Insel Riems, Germany.

### RNA-extraction

Viral RNA of swab samples was either extracted manually using the QIAmp® Viral RNA Mini Kit (QIAGEN, Hilden, Germany) or, automatically using the KingFisher™ Flex Purification System with the NucleoMag® VET Kit (Macherey–Nagel GmbH & Co. KG, Dueren, Germany). Automatic extraction was preferably used for large sample contingents. Both Kits were used according to the manufacturer’s instructions and the RNA was eluted in 100 µl elution buffer. RNA was stored at − 20 °C until further use.

### Molecular testing: reverse transcriptase real-time PCR

Samples were individually analysed for RNA specific for influenza A virus by a generic Matrix (M)-gene specific RT-qPCR based on Spackman et al., 2002 [44] as modified by [30]. Influenza A virus positive PCR samples with cq values ≤ 34 were further subtyped with modified multiplex Hemagglutinin (HA)-and Neuraminidase (NA)-specific RT-qPCRs as described by Graaf-Rau et al., 2023 [30]. The Ag-Path-ID™ One-Step RT-PCR Kit (Thermo Fisher Scientific, United States) has been used for all TaqMan based RT-qPCRs and cycling was performed on a CFX 96 RealTime PCR detection system (BIO-RAD, Germany). Samples were considered positive at a threshold cq of < 40. Samples with cq ≤ 35 were interpreted relevant to infection dynamics due to their increased viral RNA load signaling acute productive infectionand their potential to infect other pigs. This is in line with a study where virus isolation in cell culture of samples with a cq > 35 was generally unsuccessful [56], indirectly indicating lower to no infectiousness.

### Virus isolation in cell culture

Madin-Darby canine kidney-2 cells (MDCK-2, FLI Collection of Cell Lines in Veterinary Medicine CCLV-RIE 1061) or a cell line of swine testicular cells (ST cells, FLI Collection of Cell Lines in Veterinary Medicine CCLV-RIE 1061) were grown in Eagle’s Minimal Essential Medium (EMEM) supplemented with 5% fetal calf serum (FCS) in culture flasks at 37 °C in a 5%CO_2_ atmosphere. Supernatant of influenza-positive polymerase chain reaction (PCR) swab samples (cq ≤ 32) was centrifuged at 750 g for 2 min at 8 °C. For virus isolation, medium was removed from cells growing in a 25 cm^2^ culture flask and 200 µl of supernatant was added and incubated for 1 h at 37 °C in a 5%CO_2_ atmosphere. After incubation EMEM medium without FCS but supplemented with 2 µg/ml of L-1-Tosylamide-2-phenylethyl-chloromethyl-ketone (TPCK)-treated trypsin (Sigma Aldrich, USA) was added and incubated three to five days at 37 °C in a 5%CO_2_ atmosphere. Development of cytopathic effect (CPE) was observed. A further passage was started using supernatant after a freeze–thaw cycle of the previous passage. Reverse transcriptase real-time (RT-qPCR) as described below in addition to detection of a CPE confirmed successful virus isolation.

### Sequencing

Selected positive samples with cq ≤ 30 underwent whole genome sequencing by an earlier described nanopore-based amplification method by King et al., 2020 [63]. Final assembly of the sequences was done manually in Geneious Prime (Biomatters). Data have been deposited in the EpiFlu™ GISAID database (www.gisaid.org/), respective accession numbers can be found in the Supplementary file (Additional file 1: Table S2). Genotypes were assigned as previously described by Graaf-Rau et al., 2023 [30]. Hemagglutinin sequences were classified into clades using the OctoFlu tool implemented by selecting “Subspecies Classification” (https://www.bv-brc.org/app/SubspeciesClassification).

### Genotyping

Genotyping using whole genome sequences of swIAV isolated in the five German pig herds was conducted. The genotype nomenclatures was assigned as described previously by Graaf-Rau et al., 2023 [30].

### *Molecular *in silico* analyses*

The neural network-based algorithm NetNGlyc 1.0 (https://services.healthtech.dtu.dk/services/NetNGlyc-1.0/) was used to assess the N-linked glycosylation sites for the HA-1 protein of swIAV sequences gained within this study. The only motifs that were deemed possibly glycosylated were those with an N-glycosylation potential > 0.5 (threshold). According to Sun et al. 2020 [64], neutralization-relevant epitopes in the deduced HA-1 protein of swIAV sequences of two investigation series within three herds were compared. MAFFT alignments were produced with Geneious Prime (2023.0.4).

### Serological testing

Clotted blood samples were centrifuged (2000 g, 14 °C, 10 min, Heraeus Multifuge 1 S-R) and serum tested individually by a commercial ELISA kit specific for influenza A virus antibody (IDScreen® Influenza A Nucleoprotein Swine Indirect, IDvet, Germany). Results were read by Tecan infinite F200 pro and Tecan Spectra Mini ELISA readers at 450 nm, respectively. Due to its vulnerability to antigen selection and the fact that sows in all farms had experienced frequent re-vaccinations with multivalent vaccines, we decided not to carry out HI assays.

### Questionnaire

The farmer and the herd’s veterinarian were interviewed by the veterinarian conducting the study for epidemiological and holding management data using a standardized questionnaire and validated within the PIGIE consortium. Categories of the questionnaire were composed of the general herd description, characteristics of breeding stock and farrowing sector, quarantine and gilt management, anamnestic influenza A virus status, characteristics of the nursery, ventilation in the farrowing and nursery unit and biosecurity measures. Information (herd status (PRRSV), vaccination protocols) on other relevant respiratory pathogens of pigs on the respective herds was obtained by interviewing the respective herd’s veterinarian.

### Statistical analyses

All data were recorded in Excel, version 2016 (Microsoft Corporation, Albuquerque, USA). Statistical analysis was performed using SAS-Studio (3.81 Enterprise Edition, SAS Institute Inc., USA, 2020). Descriptive statistics were used to calculate the mean and standard deviation for the distribution of cq values ≤ 35 from the positive samples as well as for the cq values between > 35 and 39.9 to assess the level of excreted viral load. Median, mean values and standard deviation of cq values of samples fully subtyped and subtyped incomplete or not at all were analysed using descriptive statistic. The probability of subtyping a positive sample with cq ≤ 35 was tested using logistic regression. A mixed logistic regression by using the program R 4.4.1 was used to analyse whether piglets with low levels of passive antibodies at one week of age are more likely to exhibit high levels of shedding, indicated by cq ≤ 35. Therefore sows were set as random effect whereas farms where set to fixed effect.

## Supplementary Information

**Additional file 1.**

## Data Availability

Sequence dta have been deposited in the EpiFluTM GISAID database (www.gisaid.org/), respective accession numbers can be found in Supplementary (Supplemental Tab. 2).
