# Supplementary material for: Dynamic of swine influenza virus infection in weaned piglets in five enzootically infected herds in Germany, a cohort study
Source: Porcine Health Manag. 2024 Oct 1;10:36. doi: 10.1186/s40813-024-00390-w (PMC11446054; doi:10.1186/s40813-024-00390-w)
Supplement: Supplementary file 1 — Additional file 1. [file 40813_2024_390_MOESM1_ESM.docx]

**Supplemental Table 1.** Previous detection of swIAV infections in all five selected herds.

**Herd A**

| **Sampling year** | **Sample** | **Subtype** |
| --- | --- | --- |
| 2021 | nasal swabs | H1pdmN2 |
| 2020 | nasal swabs | H1pdmN2 |

**Herd B**

| **Sampling year** | **Sample** | **Subtype** |
| --- | --- | --- |
| 2022 | nasal swabs | not typeable |
| 2021 | nasal swabs | H1avN2 |
| 2016 | nasal swabs | H1avN2 |

**Herd C**

| **Sampling year** | **Sample** | **Subtype** |
| --- | --- | --- |
| 2022 | nasal swabs | H1avN1 |
| 2022 | nasal swabs | H1avN1/ H1pdmN2 |
| 2022 | nasal swabs | H1pdmN2 |
| 2022 | nasal swabs | H1avN1 |
| 2020 | lung tissue | H1avN2 |
| 2019 | nasal swab | H1avN2 |

**Herd D**

| **Sampling year** | **Sample** | **Subtype** |
| --- | --- | --- |
| 2022 | nasal swab | H1avN1 |
| 2020 | nasal swab | H1avN1 |
| 2020 | nasal swab | H1avN1 |
| 2020 | oral fluid | not typeable |

**Herd E**

| **Sampling year** | **Sample** | **Subtype** |
| --- | --- | --- |
| 2022 | nasal swabs | H1pdmN2 |
| 2021 | nasal swabs | H1pdmN2 |
| 2021 | oral fluid | H1pdmN2 |
| 2020 | nasal swab | not typeable |
| 2020 | nasal swab | not typeable |
| 2020 | oral fluid | H1avN1 |

**Supplemental Table 2.** Accession numbers of samples with whole genome sequencing, that have been deposited in the EpiFlu^TM^ GISAID database (www.gisaid.org/)

| **Accession number** | **Isolate Name** |
| --- | --- |
| EPI_ISL_17646286 | A/swine/Germany/2021AI07488/2021 |
| EPI_ISL_17646250 | A/swine/Germany/2022AI03311/2022 |
| EPI_ISL_17646251 | A/swine/Germany/2022AI03162/2022 |
| EPI_ISL_ 19009244 | A/swine/Germany/2022AI05640/2022 |
| EPI_ISL_19009247 | A/swine/Germany/2022AI06748/2022 |
| EPI_ISL_18977783 | A/swine/Germany/2023AI02004/2023 |
| EPI_ISL_18978822 | A/swine/Germany/2023AI02208/2023 |
| EPI_ISL_18977733 | A/swine/Germany/2023AI02202/2023 |
| EPI_ISL_19009289 | A/swine/Germany/2023AI08154/2023 |

**Supplemental Table 3.** Neutralization-relevant epitopes at sites Sa, Sb, Ca1, Ca2 and Cb within the HA-1 fragment of swIAV belonging to clade 1C sequences generated during this study. Positioning of epitopes is according to Sun et al., 2020 (1), relevant mutations are shown in color for the second batches (in comparison to batch 1) of three herds analysed here.

|  | | |  | | **Site Sa** | | | | | | | | | | | **Site Sb** | | | | | | | |
| --- | --- | --- | --- | --- | --- | --- | --- | --- | --- | --- | --- | --- | --- | --- | --- | --- | --- | --- | --- | --- | --- | --- | --- |
| **Clade** | **Herd** | **Batch** | **Age (weeks)** | **HA-1** | **124** | **125** | **155** | **157** | **159** | | **160** | **162** | **163** | **164** | **153** | | **156** | | **185** | **189** | **190** | **193** | **195** |
| **1C.2.2** | A | 1 | 10 | A/swine/Germany/2021AI07488/2021 | P | D | N | S | P | | K | R | K | S | K | | N | | E | Q | A | Q | N |
|  |  | 2 | 5 | A/swine/Germany/2022AI03311/2022 | P | **N** | N | S | P | | K | R | K | S | K | | N | | **D** | **Q** | **T** | Q | N |
|  | B | 1 | 5 | A/swine/Germany/2022AI03162/2022 | P | N | N | S | P | | K | R | K | S | K | | N | | D | Q | T | Q | N |
|  |  | 2 | 6 | A/swine/Germany/2022AI05640/2022 | P | X | N | S | P | | K | R | K | S | K | | N | | D | Q | T | Q | N |
|  | E | 1 | 6 | A/swine/Germany/2023AI02208/2023 | P | N | G | S | P | | K | K | K | S | K | | N | | D | Q | T | Q | N |
|  |  | 2 | 8 | A/swine/Germany/2023AI08154/2023 | P | N | **N** | S | P | | K | R | K | S | K | | N | | D | Q | T | Q | N |
|  |  |  |  |  |  |  |  |  |  | |  |  |  |  |  | |  | |  |  |  |  |  |
|  | | |  | | **Ca1** | | | | | **Ca2** | | | | | | | | **Cb** | | | | | |
| **Clade** | **Herd** | **Batch** | **Age (weeks)** | **HA-1** | **166** | **170** | **204** | **237** | **135** | | **137** | **140** | **142** | **221** | **222** | | **70** | | **71** | **73** | **74** | **75** | **115** |
| **1C.2.2** | A | 1 | 10 | A/swine/Germany/2021AI07488/2021 | T | G | S | G | A | | S | G | N | R | E | | L | | L | A | N | S | E |
|  |  | 2 | 5 | A/swine/Germany/2022AI03311/2022 | T | G | S | G | A | | S | G | N | R | E | | L | | L | A | N | S | E |
|  | B | 1 | 5 | A/swine/Germany/2022AI03162/2022 | T | G | S | G | D | | S | G | N | R | E | | L | | L | A | N | S | E |
|  |  | 2 | 6 | A/swine/Germany/2022AI05640/2022 | T | G | S | G | **A** | | S | G | N | R | E | | L | | L | A | N | S | E |
|  | E | 1 | 6 | A/swine/Germany/2023AI02208/2023 | T | G | S | G | A | | S | G | N | R | E | | L | | L | A | N | S | E |
|  |  | 2 | 8 | A/swine/Germany/2023AI08154/2023 | T | G | S | G | A | | S | G | N | R | E | | L | | L | A | N | S | E |

**Supplemental Table** **4.** Analysis of N-linked glycosylation sites in the HA1 fragment predicted by NetNGlyc 1.0 of sequences from each two batches of the three pig herds under investigation in this study. X indicates presence of site, x in bold indicates the addition of potential sites from batch 1 to batch 2.

|  | **herd A** | | **herd B** | | **herd E** | |
| --- | --- | --- | --- | --- | --- | --- |
| **Asparagin position on HA1 fragment** | **batch 1** | **batch 2** | **batch 1** | **batch 2** | **batch 1** | **batch 2** |
| 11 | x | x | x | x | x | x |
| 23 | x | x | x | x | x | x |
| 87 | x | x | x | x | x | x |
| 119 | - | - | - | - | - | - |
| 125 | - | - | - | - | - | - |
| 160 | - | - | - | - | - | - |
| 185 | - | - | - | - | - | - |
| 195 | - | - | - | **x** | - | - |
| 269 | - | - | - | - | - | - |
| 274 | x | **x** | - | **x** | - | - |
| 276 | - | - | - | - | - | - |
| 287 | - | - | - | - | - | - |

1. Sun H, Xiao Y, Liu J, Wang D, Li F, Wang C, et al. Prevalent Eurasian avian-like H1N1 swine influenza virus with 2009 pandemic viral genes facilitating human infection. Proc Natl Acad Sci U S A. 2020;117(29):17204-10.
